# An *IL17RA* frameshift variant in a Holstein cattle family with psoriasis-like skin alterations and immunodeficiency

**DOI:** 10.1186/s12863-020-00860-4

**Published:** 2020-05-24

**Authors:** Irene M. Häfliger, Marlene Sickinger, Mark Holsteg, Leif M. Raeder, Manfred Henrich, Siegfried Marquardt, Cord Drögemüller, Gesine Lühken

**Affiliations:** 1 Institute of Genetics, Vetsuisse Faculty, University of Bern, 3001 Bern, Switzerland; 2 Clinic for Obstetrics, Gynecology and Andrology of Large and Small Animals with Ambulatory Service, Faculty of Veterinary Medicine, Justus-Liebig University Giessen, 35392 Giessen, Germany; 3 Bovine Health Service, Chamber of Agriculture of North Rhine-Westphalia, 59505 Bad Sassendorf, Germany; 4 Institute of Veterinary Pathology, Faculty of Veterinary Medicine, Justus-Liebig-University Giessen, 35392 Giessen, Germany; 5 Veterinary Sharing Practice, Dr. Siegfried Marquardt and Peter Walter, 47574 Goch, Germany; 6 Institute of Animal Breeding and Genetics, Faculty of Agricultural Sciences, Nutritional Sciences and Environmental Management, Justus-Liebig University Giessen, 35390 Giessen, Germany

**Keywords:** Cattle, Genetic disorder, Monogenic, Mendelian, Skin disorder, Precision medicine, Rare disease, Interleukin 17 receptor a

## Abstract

**Background:**

Skin lesions and dermatoses in cattle are often associated with infections due to bacteria, fungi or environmental risk factors. Dermatoses with genetic etiology have been described in cattle. Among these rare disorders, there are primary congenital dermatoses that are associated with inherited nutritional deficiencies, such as bovine hereditary zinc deficiency or zinc deficiency-like syndrome. This study presents three cases of Holstein cattle with congenital skin lesions observed on a single farm that resemble zinc deficiency-like syndrome. Close clinical and pathological examinations took place in two cases. Pedigree analysis indicated autosomal recessive inheritance and whole-genome sequencing of both affected calves was performed.

**Results:**

The two calves showed retarded growth and suffered from severe ulcerative dermatitis with hyperkeratosis, alopecia furunculosis and subcutaneous abscess formation. Blood analysis showed correspondent leukocytosis with neutrophilia whereas minerals, macro- and micronutrients were within the reference ranges. Variant calling and filtering against the 1000 Bull Genomes variant catalogue resulted in the detection of a single homozygous protein-changing variant exclusively present in both sequenced genomes. This single-nucleotide deletion in exon 3 of *IL17RA* on bovine chromosome 5 was predicted to have a deleterious impact on the encoded protein due to a frameshift leading to a truncated gene product. Genotyping of the affected cattle family confirmed recessive inheritance.

**Conclusions:**

A loss-of-function mutation of the IL17RA transmembrane protein could be identified as most likely pathogenic variant for the psoriasis-like skin alterations observed in the two affected Holstein calves. In man, rare recessive diseases associated with *IL17RA* include immunodeficiency 51 and chronic mucocutaneous candidiasis. This supports the observed immunodeficiency of the presented cases. This study reports the first naturally occurring *IL17RA*-associated animal model.

## Background

An intact coat and skin is of utmost importance for the protection of the body from environmental impacts, including infections and thermal challenges. Clinical dermatological conditions vary in their dimensions, from alopecia, hypo- and hyperkeratosis, coat discoloration, seborrhoea to pruritus. Often several symptoms occur simultaneously. Dermatoses are reported frequently and can be due to a variety of causes. The aetiology of most skin disorders is based on a combination of genetic and environmental factors representing multifactorial or complex diseases. Nevertheless, some are determined exclusively by the environment, e.g. due to infections with pathogens belonging to bacteria, viruses, fungi and parasites [1–4], intoxication [5] and dysfunctional housing [6]. Skin lesions, e.g. due to injuries are at great risk for secondary infections. Generally, they can lead to a reduced animal welfare, performance and therefore to a loss of the economic value of an animal. In cattle, the most common and frequently endemic skin disorder in Central Europe is due to infection with dermatophytes [7]. More rarely, dermatoses occur that are determined exclusively by genetic factors, such as e.g. epidermolysis bullosa [8], or that are associated with inherited disorders leading to nutritional deficiencies of e.g. vitamin A and C, zinc, copper, or fatty acids due to malabsorption [9]. Genetic diseases of the skin are called genodermatoses and typically follow a monogenic mode of inheritance, which means that the genotype at a single gene determines whether the trait is expressed or not [10]. A hallmark of genodermatoses is familial clustering of cases and recently molecular veterinary genetics has made significant advances in the analysis of hereditary dermatoses [10].

A known genetic disorder segregating in the Holstein breed and leading to recurrent bacterial infections, delayed wound healing and retarded growth is the bovine leukocyte adhesion defect (BLAD; OMIA 000595–9913) [11, 12]. This defect is due to the p.Asp128Gly missense variant in the *ITGB2* gene [11]. The clinical symptoms induced by this variant are lesions and inflammations on oral mucous membranes and teeth, along with chronic pneumonia and diarrhoea [12].

Another autosomal monogenic recessive condition primarily seen in Holstein and Fleckvieh dairy cattle with psoriasis-like skin lesions in young calves was suspected to be the consequence of a secondary zinc deficiency [13]. It was assumed that a congenital gastrointestinal zinc malabsorption leads to an impaired function of the immune system, growth retardation and severe skin alterations (acrodermatitis). In Holstein cattle this disorder is known as bovine hereditary zinc deficiency (BHZD, OMIA 000593–9913) for a long time and reported to be associated with a recessively inherited splice-site variant in the bovine *SLC39A4* gene [14]. The altered SLC39A4 protein is described to lack two motifs, which lie in adjacent transmembrane domains involved in the construction of a pore responsible for the transport of zinc. Therefore the reported *SLC39A4* variant is most likely responsible for the impaired zinc absorption in this disorder [14]. The identification of another pathogenic variant associated with the so-called zinc deficiency-like (ZDL) syndrome (OMIA 001935–9913), a highly similar genodermatosis occurring in Fleckvieh cattle indicated obvious locus heterogeneity for this entity of disorders. Clinically a massive hyperkeratosis in combination with secondary microbial infections were observed in the ZDL-affected animals carrying two copies of the unravelled nonsense mutation in bovine *PLD4* [15]. In addition, all BHZD as well as ZDL cases were also underdeveloped in body size and weight and had a history of recurring diarrhoea and pneumonia [13–16]. Although there is a striking similarity in the phenotypic appearance of these two disorders, there are no clues for a functional connection between PLD4, an uncharacterized member of the family of phospholipid signalling enzymes, and zinc metabolism. As the ZDL-affected Fleckvieh calves did not respond to a dietary zinc supplementation, an impaired zinc metabolism is most likely not causing this disorder.

Herein we report the detailed phenotype of three half-sib purebred Holstein calves with severe congenital psoriasis-like skin alterations, retarded growth and an enhanced susceptibility to different infectious diseases such as bronchopneumonia. Clinical, genealogical, pathological and histopathological examination of the calves suggested a rare and recessive genodermatosis resembling ZDL. However, ZDL has not been observed in Holstein cattle until now. Our genetic analysis revealed a perfectly linked recessively inherited frameshift variant in the *interleukin 17 receptor A* (*IL17RA*) gene, which is most likely disease causing.

## Results

### Clinical findings

Initially, three calves were presented to the local veterinarian for clinical examination and therapy. One of the calves suffered from a severe bronchopneumonia and died shortly after presentation despite immediate therapy. The two other calves (3 and 5 months old) showed retarded growth and development even though they had been clinically inconspicuous within the first 4 to 6 weeks of their lives. Chronic skin lesions with signs of severe inflammation were present especially in skin folds, beneath the tail and at the umbilical region. Blood analysis including zinc supply, examinations for infections with bluetongue virus (BTV) or lumpy skin disease (LSD) resulted unsuspicious.

**Fig. 1:**
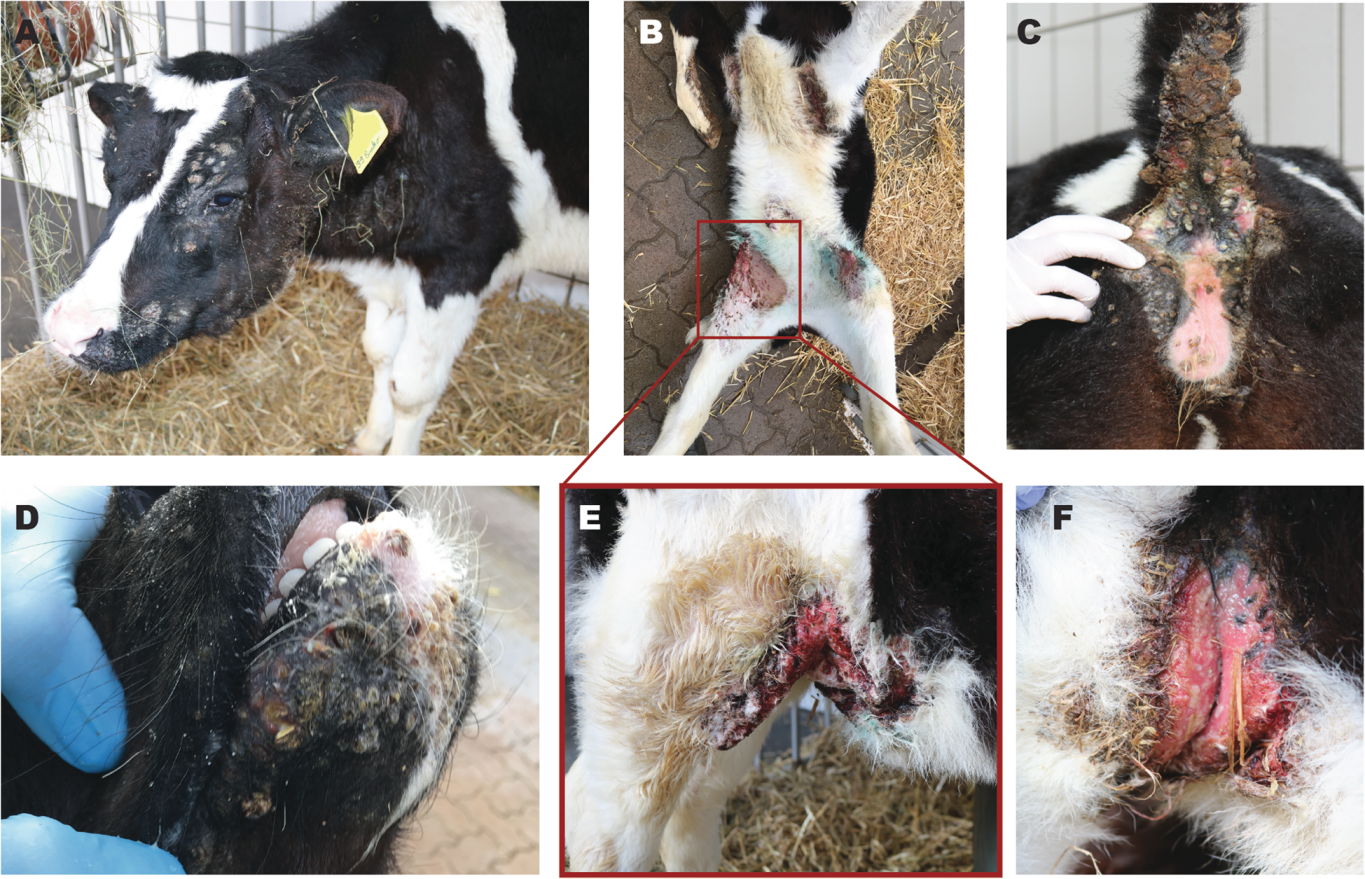
Features of the skin anomalies in case 1 (A) and case 2 (B-F). **a** Head with massive swelling at the left mandibular angle. Palpation of this mass displayed fluctuation and severe phlegmon of the skin. **b** Ventral view on the lesions in the inguinal skin. **c** Abscesses beneath the tail. Crusts of pus and incrusted faeces are present. **d** Lesions around the mouth. **e** Right inguinal skin fold with massive inflammation and ulceration. **f** Axillar skin lesions. Palpation of the inflamed skin resulted in instant bleeding

These two female calves were admitted to the Clinic for Ruminants (Internal medicine and Surgery) of the Justus-Liebig University Giessen for a deeper clinical investigation. The 5-months old calf will further be referred to as case 1 and the 3-months old calf as case 2. Both cases were lethargic and slightly dehydrated and showed massive hyperkeratotic alterations of the skin with regional alopecia and signs of inflammation. Skin lesions were partly bleeding and a sanious odour of the skin could be noticed (Fig. 1). In case 1, the head was asymmetric due to severe swelling in the region of the left mandibular lymph nodes (Fig. 1a). It was a 20 × 20 × 10 cm large mass in front of the shoulder joint. This calf also showed inflammations at the inguinal skin fold. Case 2 mainly showed marked inflammation of the axillar skin (Fig. 1f) and at the inguinal skin fold (Fig. 1b and e) as well as small abscesses at the lower surface of the tale (Fig. 1) and jaw (Fig. 1d). The palpable lymph nodes were markedly swollen in this calf.

Results of the peripheral blood analysis regarding the standard parameters are summarized in additional file 1 for each calf individually. The numbers of leukocytes, segmented neutrophils and the lymphocytes deviated from the normal range, indicating inflammation. The value for glutathione peroxidase indicated a sufficient supply of selenium in both calves. Regarding the minerals, solely magnesium showed a considerably reduced serum concentration, while zinc showed a mildly increased concentration.

Furthermore, the microbiological analysis of skin biopsies and lung tissue samples are provided in additional file 2 for both calves. Comparing the levels of infection, a high content of specific bacteria was more often observed in case 2 than in case 1, and comparing the tissue types, the bacterial content on skin was slightly higher than in the lungs. No fungal pathogens were determined in skin or lung samples. Tests concerning bovine virus diarrhoea (BVD), bluetongue virus (BTV) and bovine poxvirus (BPV) resulted negative.

### Necropsy

**Fig. 2:**
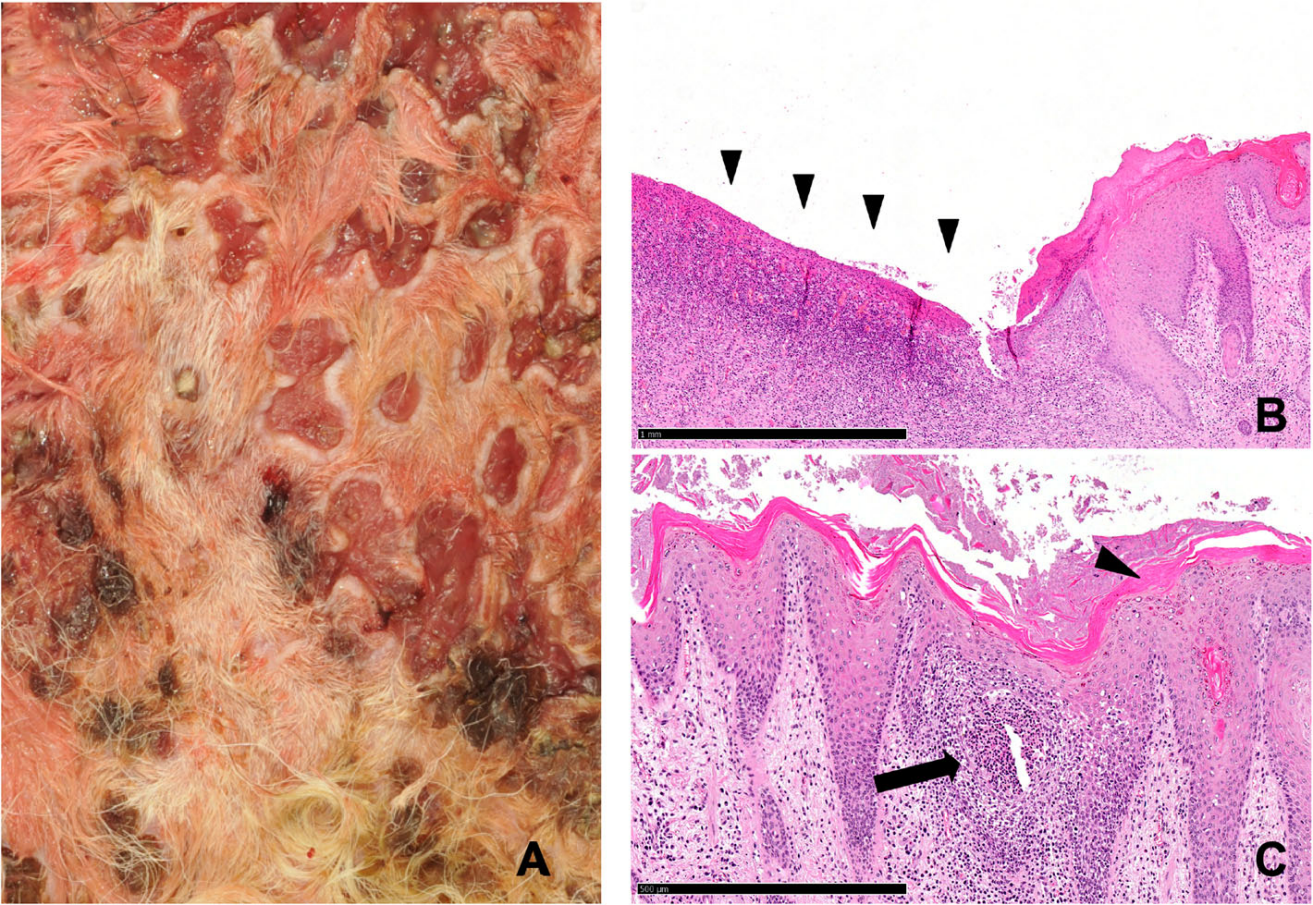
Details of the inguinal skin lesions of case 2. **a** Macroscopic image of chronic ulcerations with serocellular crusts. **b** Microscopic view of severe ulceration of the epithelium (arrowheads) with formation of granulation tissue and necrosuppurative inflammation. Hematoxylin and Eosin, 5x, bar = 1 mm. **c** Microscopic view showing marked orthokeratotic hyperkeratosis (arrowhead). Hematoxylin and Eosin, 10x, bar = 500 μm

**Fig. 3:**
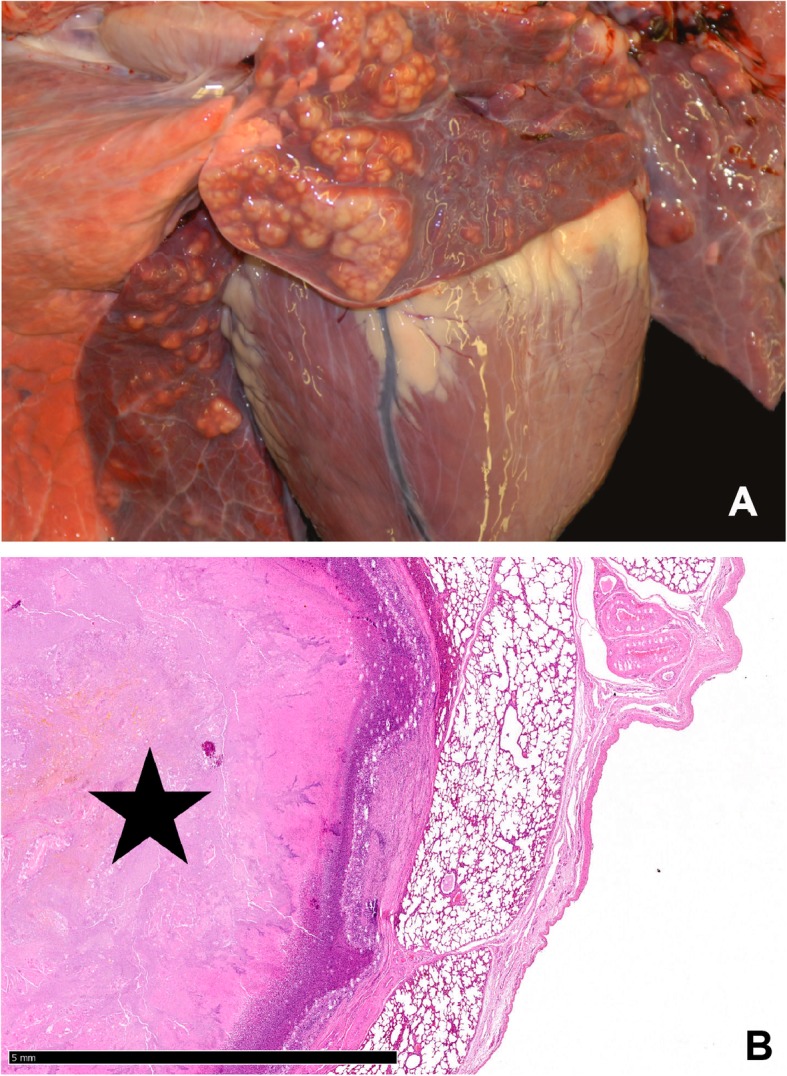
Lung of case 1. **a** Macroscopic image of multiple abscesses in the cranial pulmonary lobes. **b** Microscopic view of the abscess of the cranial pulmonary lobes (star). Hematoxylin and Eosin, 1.25x, bar = 5 mm

**Fig. 4:**
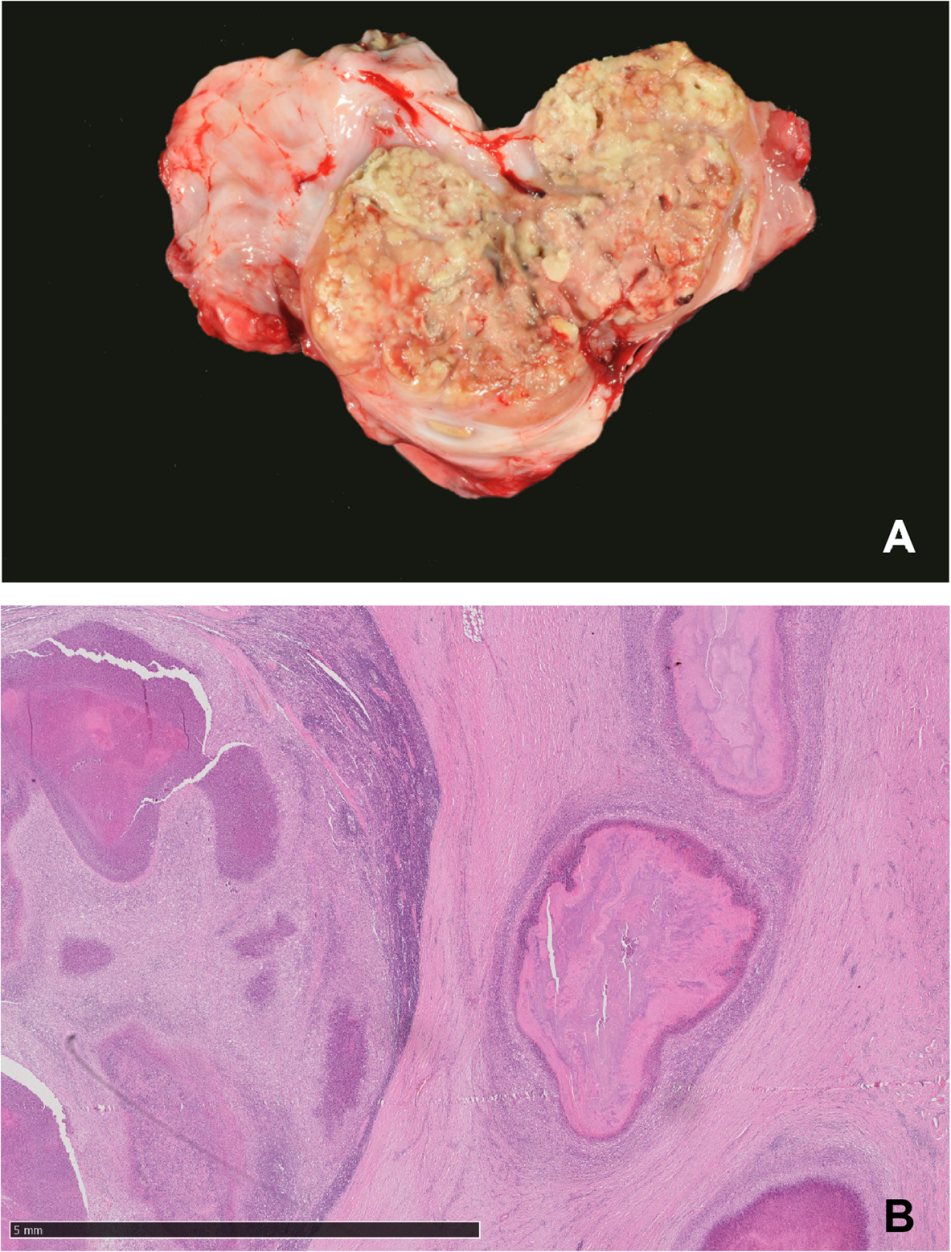
Submandibular abscess observed in case 1. **a** Macroscopic image of abscess formation at the left mandibular area, orange-sized abscess in the subcutis. **b** Microscopic view showing surrounding granulation tissue, Hematoxlyin and Eosin 1,25x, bar =5 mm

Both calves showed similar gross lesions and a deferred development with a body weight of 113 kg (case 1) and 95 kg (case 2). The dermis revealed an ulcerative dermatitis and pododermatitis with pustules, alopecia, furunculosis, subcutaneous abscess formation, and marked orthokeratotic hyperkeratosis (Fig. 1a-f). Mainly the areas of the axillar skin (Fig. 1f), the inguinal skin (Fig. 1b,e, Fig. 2a), the tale (Fig. 1c) and the mucocutaneous junctions were affected. Additionally, there was a severe epithelial hyperplasia and a moderate perivascular infiltration with lymphocytes, plasma cells, and macrophages (Fig. 2). Microscopically the massively inflamed skin lesions at the skin folds are visualized in Fig. 2b and c, where the chronic ulcerations with serocellular crusts are visible (Fig. 2a). The junctions show ulcerations of the epithelium with formed granulated tissue and the inflamed cells (Fig. 2b) and orthokeratotic hyperkeratosis with pustules were detected (Fig. 2c). Furthermore, the calves had multiple large abscesses in the cranial pulmonary lobes (Fig. 3a). They were accompanied by dystelectasis and a mild suppurative bronchopneumonia in the surrounding lung tissue (Fig. 3). There were subcutaneous masses in the region of the left mandibular lymph node and in front of the shoulder joint (Fig. 1a), and were identified as abscesses (Fig. 4).

### Genetic findings

**Fig. 5:**
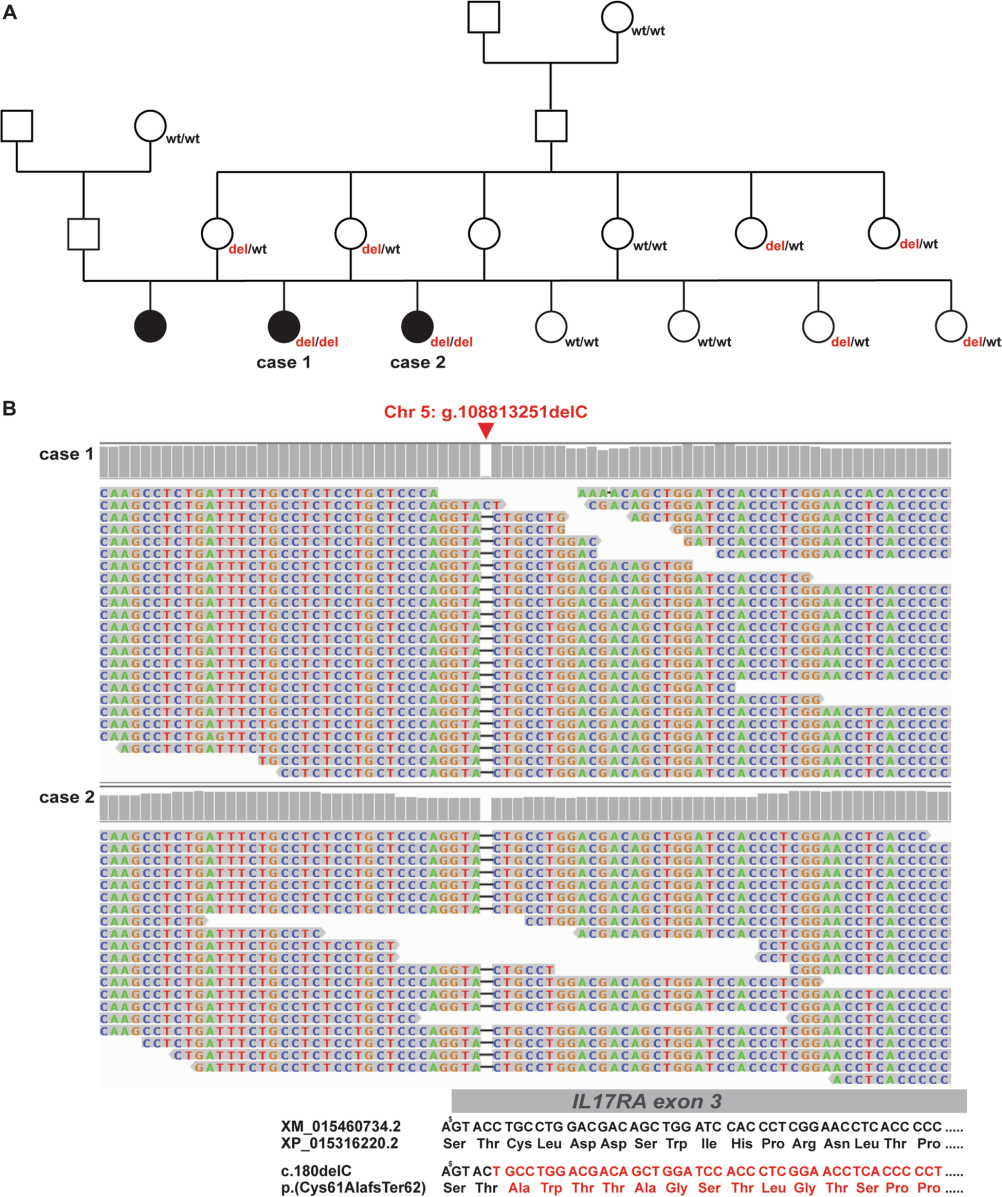
*IL17RA* frameshift variant associated with the observed genodermatosis. **a** Pedigree including the three affected calves (shown in black). All thirteen available family members were genotyped for the *IL17RA* variant (genotypes presented below the individuals). **b** IGV screenshot showing the single-nucleotide deletion on chromosome 5 in the two sequenced cases. Note the indicated change in the deduced amino acid sequence below. A premature stop codon is predicted to occur after 62 altered residues. (^**$**^ indicates the first nucleotide of codon 59 belonging to *IL17RA* exon 2)

On the farm, the sire of the three affected calves was used in a single breeding period for natural mating with a total of 30 heifers, all originating from a common natural service sire used at the farm in the years before (Fig. 5a). Besides the two affected female calves investigated in this study, 11 more female and 17 male calves were born. One of these additional female calves showed similar signs as observed in the two presented cases and died of pneumonia. However, in this additional case no close clinical examination was done and no sample is available. Neither the 17 males nor the other 10 female offspring showed comparable skin alterations although it was not possible to follow-up the health status of the male calves after they left the farm with 2-weeks of age for fattening. Both, the sire of the affected calves and all 30 dams were apparently normal. The pedigree of the affected calves is in accordance with recessive inheritance. Genetic testing allowed to rule out bovine leukocyte adhesion deficiency (BLAD) as possible cause for the observed symptoms as both cases were tested negative by a commercial laboratory (IFN Schönow GMbH, Bernau).

Adopting the assumption of a possible recessive mode of inheritance, whole-genome sequencing (WGS) followed by single-nucleotide variant (SNV) and small insertion and deletion (InDel) calling resulted in 23 private protein-changing variants (Additional file 3) beside 4754 private non-coding variants (Additional file 4). Due to the strong effect of the putative genetic defect, we hypothesized that a loss-of-function variant affecting the coding sequence of a gene most likely would be responsible for the disorder. They were found to be homozygous exclusively in the two affected animals and absent or occurring heterozygous in 396 control genomes that were sequenced in the course of other ongoing projects at the Institute of Genetics. In a second step 22 variants out of these 23 protein-changing variants were found with both possible genotypes (heterozygous and homozygous) in the 3103 control genomes from the 1000 Bull Genomes project (Additional file 3) [17]. The single remaining protein-changing variant in the *interleukin 17 receptor A* (*IL17RA*) gene, which was absent from all controls was predicted to have a deleterious impact on the encoded protein. This single nucleotide deletion in exon 3 of *IL17RA* (chr5: g.108813251delC; Fig. 5b) leads to a frameshift (XM_015460734.2: c.180delC), which is predicted to replace 61 amino acids and to truncate a significant part (~ 85%) of the C-terminus of the protein (XP_015316220.2: p.(Cys61AlafsTer62)).

We genotyped the single-nucleotide deletion in exon 3 of *IL17RA* in both available cases, as well as in eleven non-affected relatives (Fig. 5a) by Sanger sequencing. This confirmed the homozygous state of the variant in the two affected calves, whereas both dams of the affected calves were heterozygous for the *IL17RA* deletion, as well as two half-sibs and two maternal sisters (Fig. 5a). All further tested relatives were homozygous wild type.

## Discussion

Interestingly our study revealed *interleukin 17 receptor A* (*IL17RA*) as a new gene involved in a recessively inherited genodermatosis of Holstein cattle. The herein described clinical findings resemble strongly a disorder known in Fleckvieh cattle as zinc deficiency-like (ZDL) syndrome associated with a *PLD4* missense variant [15]. Both herein described cases and the ZDL-affected calves show an increasing distribution and severity of skin lesions over time, where the first lesions appeared on the muzzle. Further common characteristics are the occurrence of pneumonia and the inability to response to oral zinc supplementation [16]. However, the *PLD4* variant and the previously reported *SLC39A4*-associated zinc deficiency in Holstein cattle (BHZD) could be ruled out due to their clinical differences. While the dermatological changes associated with the *PLD4* non-sense variant are described as a crusting dermatitis [16], the herein described cases show open wounds that scarcely heal. BHZD affected calves show a zinc deficiency, which can be treated by oral application of zinc [13]. This therapy was applied to the herein presented cases but did not lead to any clinical improvement and thereby a disorder like BHZD could be ruled out. Furthermore, bovine leukocyte adhesion deficiency (BLAD) was excluded through genetic testing. Finally, all known disease-causing variants were confirmed to not be present in the described cases by analysing the WGS data.

To our knowledge, the *IL17RA* gene had not yet been associated with similar disorders in domestic animals. Variants in human *IL17RA* were reported to cause autosomal recessively inherited immunodeficiency 51 (OMIM 605461) and affected individuals show inborn susceptibility to several infections and especially to chronic mucocutaneous candidiasis [18, 19]. The pathogenic variants associated with immunodeficiency 51 include missense variants as well as non-sense variants and lead to a total loss-of-function of IL17RA [19, 20]. Pro-inflammatory cytokines such as interleukin 17 and 22, as well as their receptors play a central role in acute and chronic inflammatory responses [21, 22]. Interleukin 17 (IL-17) is especially known to be of importance at mucosal and barrier surfaces and plays a possible role in the pathogenesis of autoimmunity [23]. The most common member of the IL-17 family is IL-17A and the receptor complex it signalling threw the heterodimer complex formed by the receptors IL-17RA and IL-17RC [23–25]. Furthermore, IL-17 had been associated with a wide range of diseases including inflammation ranging from arthritis, psoriasis, spondylitis, Crohn’s disease, multiple sclerosis, cardio vascular diseases and a variety of disorders of the lung [23]. The involvement of IL-17 and its receptor IL17RA has been shown in respect to psoriasis and psoriatic arthritis in humans and mice [26–30].

Interestingly, the herein described bovine cases homozygous for the *IL17RA* loss-of-function variant did not suffer from fungal but from multi-bacterial infections. This points to differences in the immune defence against fungal and bacterial infections between humans/mice and cattle.

This *IL17RA*-associated semi lethal genodermatosis, leading to psoriasis-like skin alterations and immunodeficiency resembling zinc deficiency-like syndrome in Holstein cattle, was not reported before. Based on the absence of the disease-causing *IL17RA* variant in the global cohort of whole-genome sequenced cattle, which includes several hundred Holstein sires used in artificial insemination, the frequency of the mutant allele in the international Holstein population is probably very low. Nonetheless, our findings allow for the first time a targeted monitoring of the prevalence of this most likely pathogenic variant in the local German population and the avoidance of further risk matings.

## Conclusion

To the best of our knowledge, a similar *IL17RA*-associated genodermatosis showing chronic skin alterations correlated with an inherited immunodeficiency has not been described until now in cattle. Moreover, this is the first report of a most likely pathogenic variant in *IL17RA* in a domestic animal species.

## Methods

### Animals

In a single German Holstein breeding flock three calves which were sired by the same bull showed clinical signs suspicious for a disease similar to ZDL or BHZD. While one calf perished of a heavy lung inflammation during the first weeks of life the other two calves were chronically suffering from psoriasis-like skin alterations and showed reduced growth. Therefore, in this study clinical and pathological examinations were conducted on the two affected calves and the genetic background. Blood samples from eleven healthy relatives were collected. Unfortunately, the sire was already slaughtered when the study was executed. In addition, we collected blood of two dams, the paternal grandmother, the maternal great-grandmother, four paternal half-sibs and three paternal half-sibs of the dams (Fig. 5a).

### Clinical analyses

Clinical examination was conducted on two affected calves. This includes a general description of their health status. Further, all important peripheral blood parameters were analysed. Blood analyses were performed using EDTA, serum and lithium-heparin samples from each of the affected calves. After necropsy microbiological analysis of the skin and lung tissue had been performed.

### Whole-genome sequencing

In order to investigate the genetic architecture of the disorder, genomic DNA was extracted from EDTA blood samples of case 1 and 2 and used for whole-genome sequencing (WGS). Therefore, individual PCR-free fragment libraries, which were sequenced for 150 bp paired-end reads were prepared. Both cases were sequenced on the Illumina NovaSeq6000 resulting in a read depth of approximately 17.2x and 20.8x for the first and the second case, respectively. The WGS data was mapped to the latest reference genome ARS-UCD1.2 [31] and called for small nucleotide variants (SNVs) and small insertions and deletions (InDels). We followed the workflow proposed by the 1000 Bull Genomes Project (run 7) [17, 32] to process the raw data into binary alignment map (BAM) and genomic variant call format (GVCF) files. Further, the individual GVCF files were merged to one large variant call format (VCF) file by using CombineGVCFs and CatVariants of GATK v3.8 [33]. This file was produced together with another 396 genomes available at the Institute of Genetics of the University of Bern and thereby includes 398 genomes (Additional file 5) of a variety of 23 cattle breeds and few crossbred animals. For this VCF file SNVs and InDels were called using GenotypeGVCF of GATK v3.8 [33]. Furthermore, quality labels based on the best practice recommendations in GATK using the VariantFiltration of GATK v3.8 [33] were given for each variant. Finally, SnpEff v4.3 [34] was used to functionally annotated each variants effect, based on the NCBI Annotation Release 106 [35]. This resulted in the final VCF file, which includes all 398 individuals’ variants and their functional annotation.

We filtered the final VCF file based on the assumption that the disorder is a rare recessive disorder. Thereby, only the two affected animals need to be carriers of a possible candidate variant but no other of the 396 control genomes (Additional file 5). This approach is supported by the fact, that there are no other sequenced German Holstein animals in the data set. In order to validate the allele frequency of the variant and exclude variants that occur in other breeds all remaining variants were compared to the VCF file from the 1000 Bull Genomes project (run 7) [17]. This reference data includes 3103 cattle genomes which include 937 individuals from the Holstein breed from all around the globe. Integrative Genomic Viewer (IGV) software [36] was used for visual inspection of the remaining candidate variants.

### Genotyping of the candidate variants

Forward primer 5′-GTCATGGCCTGACTGTGAAG-3′ and reverse primer 5′-GTCCACTCGATGTGAACCAC-3′ were designed with the software Primer3 [37] to produce a 243 bp fragment including the *IL17RA* variant. Sanger sequencing of the resulting PCR product was performed by a service laboratory (LGC Genomics, Berlin). The obtained sequences were analysed with ChromasPro 1.22 software (Technelysium Pty Ltd., South Brisbane) and compared with the relevant genome region of the latest reference genome ARS-UCD1.2 [31].

## Supplementary information

**Additional file 1.** Results of peripheral blood analyses.

**Additional file 2.** Results of microbiological analyses in lung and skin samples. Note that the analyses could not display any mycological agents.

**Additional file 3.** Twenty-three shared homozygous protein-changing variants in both sequenced cases. The frequency of the corresponding genotypes in the variant catalogue of the 1000 Bulls Genome project are given.

**Additional file 4.** 4754 shared homozygous non-coding variants in both sequenced cases.

**Additional file 5.** EBI accession numbers of all publicly available genome sequences.

## Data Availability

Whole-genome sequencing data can be accessed on the European Nucleotide Archive with the project ID PRJEB18113 (sample accessions: SAMEA5714977; SAMEA5714978). Further publicly available data used in this study can be found in this repository with the indicated sample IDs in the additional file 4.

## Abbreviations

BAM: Binary alignment map
BHZD: Bovine hereditary zinc deficiency
BLAD: Bovine leukocyte adhesion deficiency
BPV: Bovine poxvirus
BTV: Bluetongue virus
BVD: Bovine virus diarrhoea
GVCF: Genomic variant call format
InDel: Small insertion and deletion
LSD: Lumpy skin disease
SNV: Single-nucleotide variant
VCF: Variant call format
WGS: Whole-genome sequencing
ZDL: Zinc deficiency-like
